# Chromosome Inversions, Genomic Differentiation and Speciation in the African Malaria Mosquito *Anopheles gambiae*

**DOI:** 10.1371/journal.pone.0057887

**Published:** 2013-03-20

**Authors:** Yoosook Lee, Travis C. Collier, Michelle R. Sanford, Clare D. Marsden, Abdrahamane Fofana, Anthony J. Cornel, Gregory C. Lanzaro

**Affiliations:** 1 Vector Genetics Laboratory, Department of Pathology, Microbiology and Immunology, School of Veterinary Medicine, University of California Davis, Davis, California, United States of America; 2 Department of Entomology, University of California Davis, Davis, California, United States of America; 3 Malaria Research and Training Center, Faculty of Medicine, University of Mali, Bamako, Mali; National Institute for Communicable Diseases/NHLS, South Africa

## Abstract

The African malaria vector, *Anopheles gambiae*, is characterized by multiple polymorphic chromosomal inversions and has become widely studied as a system for exploring models of speciation. Near complete reproductive isolation between different inversion types, known as chromosomal forms, has led to the suggestion that *A. gambiae* is in early stages of speciation, with divergence evolving in the face of considerable gene flow. We compared the standard chromosomal arrangement (*Savanna* form) with genomes homozygous for *j*, *b*, *c*, and *u* inversions (*Bamako* form) in order to identify regions of genomic divergence with respect to inversion polymorphism. We found levels of divergence between the two sub-taxa within some of these inversions (2R*j* and 2R*b*), but at a level lower than expected and confined near the inversion breakpoints, consistent with a gene flux model. Unexpectedly, we found that the majority of diverged regions were located on the X chromosome, which contained half of all significantly diverged regions, with much of this divergence located within exons. This is surprising given that the *Bamako* and *Savanna* chromosomal forms are both within the S molecular form that is defined by a locus near centromere of X chromosome. Two X-linked genes (a heat shock protein and P450 encoding genes) involved in reproductive isolation between the M and S molecular forms of *A. gambiae* were also significantly diverged between the two chromosomal forms. These results suggest that genes mediating reproductive isolation are likely located on the X chromosome, as is thought to be the case for the M and S molecular forms. We conclude that genes located on the sex chromosome may be the major force driving speciation between these chromosomal forms of *A. gambiae*.

## Introduction

Species divergence, and the evolution of reproductive isolation among populations within a species, can be mediated by several mechanisms. One such mechanism is through chromosomal inversions. A paracentric chromosome inversion is the result of chromosome breakage and repair where a chromosome fragment that does not include the centromere, is re-inserted in reverse orientation, resulting in an inverted gene orientation. This type of inversion can have both structural effects (e.g. alteration of gene expression, chromosome anomalies generated during meiosis) and/or genetic effects (e.g. reduced recombination) [1], [2]. Reduced recombination associated with paracentric inversion heterozygotes can result in an accumulation in local populations of alleles that confer a fitness advantages or that are deleterious [3]. This occurs because inversions protect gene regions in diverging groups from the homogenizing effects of gene flow [4], [5]. Consequently, inversions may contain areas of relatively high nucleotide divergence in populations under divergent selection or in the early stages of speciation, even with some degree of gene flow between them [1], [6].

A prominent feature in the genomes of divergent or diverging populations that continue to experience some degree of gene flow is a heterogeneous or mosaic pattern of genomic differentiation [7]. Chromosomal inversions may therefore serve as a mechanism through which sympatric species diverge in the face of gene flow by protecting regions of the genome that contain genotypes that are adaptive to different microhabitats or are responsible for reproductive isolation [4]. In populations undergoing speciation with gene flow, as is thought to be the case in populations of the African malaria vector, *Anopheles gambiae,* patterns of genome heterogeneity have been identified that in some instances strongly coincide with inversion polymorphisms [8], [9].

The inversions of the *A. gambiae* complex have long been thought to be associated with adaptation to human mediated environmental change and have been credited with the success of the most capable malaria vectors in the complex, including the nominal species, *A. gambiae* sensu stricto (hereafter *A. gambiae*). [10]. The subspecific *chromosomal forms* of *A*. *gambiae* (haploid chromosome n = 3) are defined by the configuration of five paracentric inversions on the right arm of chromosome 2 (2R*j*, *b*, *c*, *d* and *u*) and one on the left arm of chromosome 2 (2L*a*). Surveys of *A. gambiae* populations where these inversions occur in sympatry have revealed a deficiency or complete absence of certain inversion heterozygotes, suggesting that there are barriers to gene flow among the chromosomal forms [10]–[12], despite a lack of evidence for post-zygotic isolation [13], [14]. The five chromosomal forms of *A. gambiae* have been named *Mopti*, *Bamako*, *Bissau*, *Forest* and *Savanna* according to the geographic regions from which they were first collected and indicating an association of each with a particular habitat [10]. Inversion frequencies have also been associated with ecological clines [12], [15], [16] and seasonal patterns of rainfall [12] contributing even more evidence for a very close relationship between inversion polymorphism and environmental parameters.

Further complicating efforts to describe the genetic structure of *A*. *gambiae* populations in West Africa was the identification of the two *molecular forms*, M and S, recognized on the basis of fixed sequence differences in an X-linked intergenic spacer of the multi-copy 28S ribosomal DNA gene [17]–[19]. Despite occurring in sympatry throughout much of West and Central Africa, hybrids between M and S are generally rare in nature [16], [17], [20], [21] with the exception of locations in The Gambia, Guinea-Bissau [22]–[24] and Burkina Faso[25]. No evidence of post-zygotic isolation between them has yet been demonstrated [26], suggesting that the mechanism responsible for the observed reproductive isolation is pre-zygotic [27], [28]. Consistent with genic models of speciation [29], [30], microsatellite analyses [31]–[34] and microarray studies [8], [35], [36] have revealed a heterogenous pattern of genomic divergence between the two molecular forms creating a mosaic, whereby differentiation is restricted to a few, relatively small regions of the genome.

The chromosomal or ecotypic model of speciation has been widely accepted to explain divergence within *A. gambiae* [37]–[39]. This model is founded on the observation that certain paracentric inversions are non-randomly distributed in nature and are thought to contain multi-locus genotypes that are adaptive to specific aquatic habitats occupied by the immature stages [38]. Under this model, populations carrying alternate gene arrangements inhabit different, spatially isolated habitats. Genetic divergence, enhanced by reduced recombination, would then evolve [6], [40], [41]. Ultimately divergence would include genes resulting in reproductive isolation (reduced fitness in hybrids or behavioral differences preventing between form mating), explaining the observed deficiency of inversion heterozygotes.

One expectation of the chromosomal (or ecotypic) theory of speciation is that for recently diverged populations differentiation will be higher in regions contained within, or linked to, inversions, relative to elsewhere in the genome. This hypothesis has been examined by White et al. [42] who used a transcriptome based *Anopheles gambiae*/*Plasmodium falciparum* array to examine divergence on chromosome 2R between inverted and uninverted M and S form genomes. Interestingly, across the four 2R inversions studied (∼26 Mb), divergence was limited to just one significantly diverged feature (SDF) in the 2R*u* inversion covering only a ∼100 kb region of the genome. This result contrasts with analysis of the 2L*a* inversion, where ∼3 Mb was found to be divergent [43]. This unexpected result was attributed to a recent emergence of the 2R*u* inversion, suggesting that insufficient time has passed for complete lineage sorting (adaptive mutations on the inversion may still be present in the standard form) and widespread divergence to evolve, while the impact of gene flux on the more ancient inversions of 2R*b* and *c* has caused divergence in these taxa to decay. The authors also suggest, that the relatively low resolution of the microarray used in their study, may have failed to detect diverged regions if they consisted of just a few genes. Lastly, they point out that it is possible that inclusion of samples from both the M and S molecular forms in the microarray comparison may have confounded divergence signals relative to chromosome inversions.

In this analysis comparing *A*. *gambiae* genomes, we utilize a high resolution custom whole genome tiling microarray (WGTM) to compare the genomes of the *Bamako* and *Savanna* chromosomal forms. The use of the WGTM provides us with several advantages over the commercially available GeneChip *Plasmodium/Anopheles* Genome Array (*P*/*A* Genome Array) used in previous studies [8], [36]. The WGTM enables examination of both coding and non-coding regions of the genome including all chromosomes at high resolution (1∶17 bp overall for WGTM vs. 1∶100,000 bp for the *P*/*A* Genome Array). The WGTM is based on the most recent *A. gambiae* sequence (completed in 2006) while the *P/A* Genome Array is based on Build 2, released in 2003. In addition, the use of the WGTM allows the examination of mechanisms of divergence and speciation such as the determination of copy number variations (CNVs). There is widespread evidence that CNVs may be a force in divergence when duplicated genes evolving completely new functions [44]–[49].

In this study, we chose to examine two chromosomal forms of *A*. *gambiae*, while controlling for the potentially confounding effect of molecular forms by examining the genomes of the *Bamako* and *Savanna* chromosomal forms both of which are S molecular forms of *A*. *gambiae*. The *Bamako* form which is largely restricted to areas adjacent to the Niger river, includes three genotypes, *jcu*/*jcu*, *jcu*/*jbcu*, and *jbcu*/*jbcu*, all homozygous for *j* [12]. Whereas the *Savanna* form has a broader distribution across the Savanna ecological zone in Africa and includes many different karyotypes but the most common arrangements are combinations of *b*, *bcu*, *cu*, and standard (+) haplotypes and lack the *j* inversion [12]. To reduce the potential for confounding effects of differential selection on inversions associated with the *Savanna* form the standard (uninverted) *Savanna* of Mali was selected for this study. This also allowed for a direct comparison of mosquito genomes strictly based on chromosomal inversions.

Given the strong evidence for a role of inversions in local adaptation, and their defining role for the chromosomal forms, most research into divergence of the chromosomal forms has concentrated on the inversions themselves [15], [35], [38]. Under the chromosomal speciation hypothesis, genes mediating reproductive isolation are expected in or near chromosomal rearrangements where recombination is suppressed [5], [6]. This allows us to make the prediction that if ecotypic speciation is important in this system divergence will be higher in regions of the genome linked to inversions compared with those that are not. If as White et al. (2009) found that divergence was not particularly elevated within inversions we can evaluate several alternative hypotheses: If divergence is not limited to the inversions where is divergence located genomically?; Is there evidence for X- and/or non-X linked chromosome islands of speciation between two chromosomal forms, as has been shown for the M and S molecular forms? (i.e. are the genes driving divergence between M and S, also important for divergence between *Bamako* and *Savanna*.) and; Is there evidence for a role of gene duplication in divergence between *Bamako* and *Savanna*?

## Results

### Genomic divergence between and within the *Bamako* and *Savanna* chromosomal forms

The goal of this study centers on a comparison of S molecular form genomes representing different gene arrangements imposed by paracentric inversions using WGTMs, with the aim of further understanding divergence in *A. gambiae* due to chromosomal inversions and genetic elements involved in reproductive isolation. To maximize the power of our comparison, we selected specimens fixed for the four 2R inversions (*Bamako* form, Table 1) for comparison with individuals lacking any of these inversions (standard arrangement, *Savanna* form in Table 1). Individuals of the S molecular form with the standard arrangement are relatively scarce in Mali (only 14 of the 1,118 *A*. *gambiae* 2L*a*/*a* samples from Mali in the *Pop*I database [50] were standard for all other inversions as of 29 June 2012). Consequently, it was necessary to utilize material collected over a four year period (2002–2006, Table 1) from multiple locations.

**Table 1:** Genetic information for mosquito samples used for microarray hybridization.

Chromosomal form	*Savanna*	*Bamako*
Molecular form (28S r DNA)	S (S/S)	S (S/S)
2L	*a*/*a*	*a*/*a*
2R	+/+	*jbcu*/*jbcu*
Sample IDs	A1: Banambani_2005-07-23_112	B1: Banambani_2005-07-23_088
	A2: Kokouna_2002-08-08_013	B2: Banambani_2006-08-10_024
	A3: Kokouna_2002-08-08_034	B3: Kela_2006-10-11_007
	A4: Selinkenyi_2006-08-17_041	B4: Kela_2006-10-11_037
	A5: Selinkenyi_2006-08-17_117	B5: Kela_2006-10-11-052
	A6: Yorobougoula_2002-11-06_081	B6: Selinkenyi_2006-08-17_014
	A7: Yorobougoula_2002-11-06_100	B7: Selinkenyi_2006-08-17_038

Sample IDs represent a concatenation of collection site, collection date and tube index number, and correspond to identification numbers stored on the publically available online database, *PopI* at: https://grassi2.ucdavis.edu/. In addition, photomicrographic images of polytene chromosome preparations used to determine karyotypes for all specimens, except the two samples from Yorobougoula, are available on *PopI*.

We first assessed divergence between the *Bamako* and *Savanna* forms by calculating genetic similarity. Specifically we used the π_0_ statistic as defined by Storey and Tibshirani [51] which describes the overall proportion of two-sample comparisons of probe intensity resulting in non significant p values using Q-value software. The maximum value of π_0_ = 100% indicates no significant genetic differentiation, while π_0_ = 0% indicates all probe intensities were significantly different between two forms. Based on this metric we found π_0_ between *Bamako* and *Savanna* genomes to be 47.9%. By comparison, when we calculated π_0_ within the *Bamako* and *Savanna* forms separately by randomly assigning the samples within each form into two groups and calculating π_0_ between these groups, we found overall π_0_ to be 100%. This indicates high genome similarity and no significant genetic differentiation among individuals within forms.

Visual inspection of a random sample of diverged regions revealed a small region (4–5 kb/230 MB) of divergence among samples within the *Savanna* form that was correlated with collection date (Figure S1). In order to explore the occurrence of temporal divergence in the *Savanna* form on the genomic scale we subdivided samples by collection date (samples A1, A4, A5 [2005–06] vs. A2, A3, A6, A7 [2002]) and calculated π_0_. However, this comparison yielded π_0_ of 100%, indicating no significant divergence between samples collected at the two time points, which suggests that genomes of the two sets are very similar.

**Figure 1:**
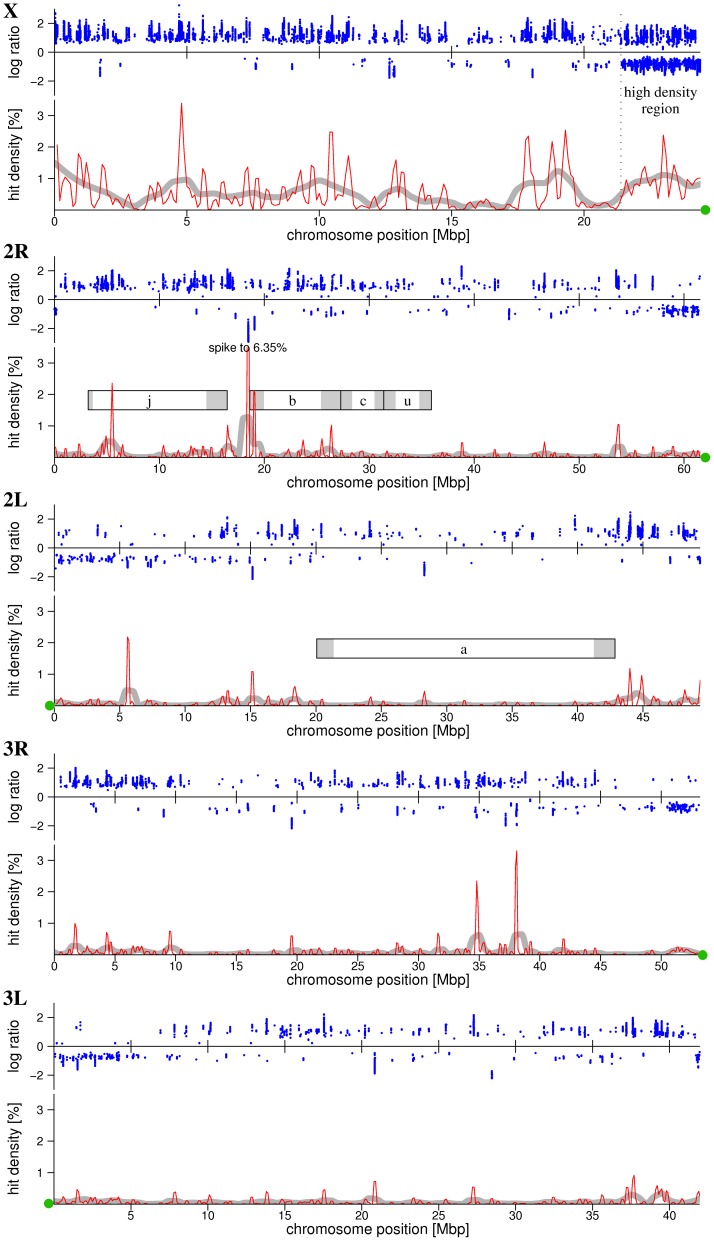
Distribution of divergence on each chromosome. Fraction of significant probes, denoted “hit density”, using a 200 kbp windows across the X, 2R and 2L chromosomes is indicated by the solid red line. Smooth gray lines indicate the fraction of significant probes in 1 Mbp windows. Positions of inversions (*j*,*b*,*c*,*u* and *a*) are depicted as rectangles, with shading indicating the approximate location of inversion breakpoints. Centromere positions are marked as green dots on the X axis. The log-ratio of significant probes is marked with blue dots, log ratios above zero are probes with higher signal intensity in the *Bamako* relative to *Savanna* forms, below zero weaker signal intensity. Log-ratio of 1 indicates a two fold increase in hybridization signal intensities in *Bamako* forms relative to *Savanna* forms. Log-ratio of −1 indicates a two fold increase in signal intensities in *Savanna* forms compared to *Bamako* forms.

Based on the π_0_ values, false discovery rates (FDR) are estimated using Q-value software. Using a FDR of 0.001, equivalent to a p-value of 1.562075×10^−6^, as the significance threshold, we detected 19,906 significant features (SFs) where the *Bamako* and *Savanna* genomes differ, as illustrated in Figure 1. Using this conservative FDR, only 20 of the 19,906 SFs could be false positive. The blue dots in Figure 1 depict a significant log ratio at each genomic location. In some areas of the genome, SFs were densely populated, resulting in a clustering of blue dots. In order to enhance visualization of these hotspots of divergence, we calculated a hit density metric, which represents the fraction of SFs per 200 kbp window, and plotted this for each chromosome arm, as illustrated by the red line in Figure 1. Lastly, we assessed the total size of significantly differentiated regions (SDRs) between the *Bamako* and *Savanna* forms (Table 2); as described in the terminology section below. Using the conservative FDR ( = 0.001) we found a total of 410 kbps (0.18% of the genome) to be significantly diverged between the *Bamako* and *Savanna* genomes (Table 2).

**Table 2:** Total size of significantly differentiated regions (SDRs) in base pairs along different chromosome segments.

Role	2R	2L	3R	3L	X	X3M	Total
Exon	19,201	2,327	3,339	2,529	3,025	2,470	**32,891**
Intron	20,983	9,743	6,240	4,722	22,961	9,356	**74,005**
Promoter	369	25	729	305	145	303	**1,876**
Terminal	350	316	544	151	1,307	30	**2,698**
Intergenic	40,623	31,396	48,414	27,620	119,636	31,322	**299,011**
Total	81,526	43,807	59,266	35,327	147,074	43,481	**410,481**
Genome size (Mbp)	61.5	49.4	53.2	42.0	21.4	3.00	**230.5**
% chr.	0.133	0.089	0.111	0.084	0.687	1.449	**0.178** *
Total # genes	**3,582**	**3,054**	**2,579**	**2,118**	**1,057**	**37**	12,427^**^
Diff. genes	110	83	64	67	80	22	**426**
Diff. genes on exon	32	33	22	31	20	19	**157**

* Total proportion of significantly differentiated regions in relation to the total *A. gambiae* genome size ( = 273 Mbp) **The remaining genes 827 ( = 13,254–12,427) belong to UNKN segment, a large segment that has not mapped to chromosome.

### Genomic regions showing divergence between *Bamako* and *Savanna* forms

**Figure 2:**
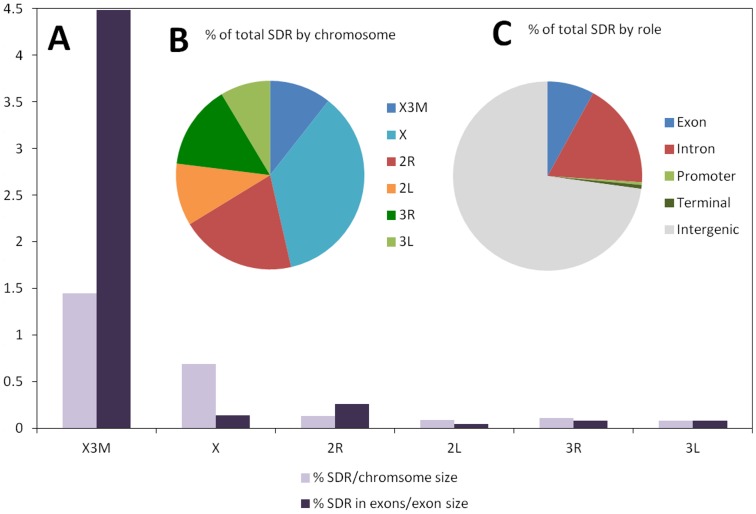
Distribution and characterization of genomic divergence. **A:** Proportion (%) of significantly differentiated regions (SDRs) expressed as a percentage of the total chromosome segment size are shown in light colored bars. Proportions of SDRs in exons as a percentage of the total exon size per segment are shown in dark colored bars. The X3M segment includes the 3 Mbp region proximal to the centromere. The X includes the X chromosome minus the X3M region. **B:** Proportion (%) of total SDR size categorized by chromosome segment. **C:** Proportion (%) of total SDR size categorized by the role of the nearest gene. An SDR is classified as 'intergenic' if there is no gene located within 250 bp from either end of that SDR.

Whilst, SFs were found on all chromosomal arms, the X chromosome had the highest proportion of SFs (Figure 1 &2, Table 2). Nonetheless, elevated differentiation was detected on 2R near the breakpoints of inversions *j* and *b* (Figure 1). On chromosome 3, very little divergence was detected on the left arm. Two divergence hot spots were identified on the right arm of chromosome 3, but these were not associated with known inversions (Figure 1). Whether this region is directly linked to reproductive isolation or divergence accumulated after behavioral isolation remains to be determined.

A similar pattern of divergence was found based on the distribution of SDRs, where the proportions of each chromosome differing significantly varied by chromosome segment (ANOVA, F = 35.246, P = 0.004; Figure 2B). It is noteworthy that if the 3 Mbp region near the centromere on the X chromosome (denoted X3M) is excluded, chromosome is no longer a significant factor contributing to the proportion of SDRs (F = 9.222, P = 0.056). We found no evidence that SDRs are concentrated within inversions (Wilcoxon rank-sum test, P = 0.82). However, as illustrated in Figure 1, there appear to be hotspots of genetic differentiation at the breakpoints of the *j* and *b* inversions. Significant divergence in the *c* and *u* inversions was minimal and was limited to non-coding regions. At the breakpoints of 2Rc, intron regions of AGAP002817, AGAP002986, and AGAP003059 were significantly diverged. At the breakpoints of 2Ru, intron regions of AGAP003059, AGAP003114, and AGAP003335 were significantly diverged. However, the density of SFs in the *c* and *u* inversions was not elevated as in the *j* and *b* inversions.

The majority of SDRs were concentrated in non-coding parts of the genome, with only 8% located within known or putative exons (Figure 2C). We tested for skewed divergence in coding regions in comparison with non-coding regions within chromosome inversions using Chi-Squared tests. All inversions except *b* had significantly more divergence in non-coding regions than coding regions (Table 3). By contrast, the X3M region of the X chromosome exhibited more divergence in coding regions than non-coding regions (Chi-squared test P<2.2e−16).

**Table 3:** Divergence in coding sequences contained in chromosome inversions.

Segment	probes in non-coding	Probes in exons	SF in non-coding	SF in coding	%SF in non-coding	%SF in exons	X-squared P value
X3M	473922	12748	4424	252	0.933	**1.977**	<2.2e−16
2R*j*	601191	121258	841	32	**0.140**	0.026	<2.2e−16
2R*b*	397543	75115	650	92	0.164	0.122	0.01075
2R*c*	185487	37963	122	1	**0.066**	0.003	3.211e−06
2R*u*	202416	39978	67	0	**0.033**	0	0.0005
2L*a*	1078072	164390	301	6	**0.028**	0.004	9.090e−09

Significantly greater proportions of significant features (SFs) in exons or non-coding regions are marked in bold.

We examined the location of each SDR with respect to the annotated genes of *A. gambiae* in order to detect any potential functional divergence between the two chromosomal forms. Only 3% ( = 425/13254) of currently annotated genes were found to overlap with SDRs, and of these, only 9 are known genes. The functions of these genes are listed in Table 4 and include two proteins that were identified to have fixed differences between the Forest-M and Forest-S chromosomal forms in a previous study [8]: a heat shock protein (AGAP001070), and cytochrome P450 (AGAP001076). Overall, the proportion of SDRs which overlap exons was higher in the X3M region than any other chromosome segment (Chi-square Test, P = 0.0002, Figure 2A). This does not appear to be due to a higher density of genes within this region because gene density is lower in the X3M region (one every 81 kbps) than other chromosome segments (one every 17–20 kbps).

**Table 4:** Selected list of known genes of which exons overlap with SDRs.

chr	gene id	Description
X	AGAP000562	Moesin/ezrin/radixin homolog 1
X	**AGAP001070**	Heat shock protein (Fragment).
X	**AGAP001076**	Cytochrome P450 CYP4G16 (Fragment).
2R	AGAP001487	Innexin shaking-B
2L	AGAP004719	Clip-Domain Serine Protease, family C
3R	AGAP008023	Segmentation polarity homeobox protein engrailed
3L	AGAP010423	JAKSTAT pathway signaling, Signal-Transducer and Activator of Transcription 1 (STAT 1)
3L	AGAP010505	Candidate odorant receptor, GPROR44
3L	AGAP011949	Caspase, CASPS 1

Bold type depicts genes shown to include fixed differences between the Forest-M and Forest-S forms of *A. gambiae* by Turner *et al.* (2005).

### Divergence and Gene/Exon Duplication

A feature or probe is determined to be significantly diverged between the *Bamako* and *Savanna* forms when the distributions of hybridization strengths, measured by fluorescent light intensities, are statistically different using a Wilcoxon rank sum test. The difference in hybridization strengths may be due to differences in DNA sequence or to differences in sequence copy number. We measured the log-ratio of median intensities between *Bamako* and *Savanna* to estimate relative enrichment. A log-ratio greater than 1 or less than −1 for multiple consecutive probes suggests that a relatively large scale mutation, like gene or exon duplication, has occurred. The expectation is that a duplication event will yield a log-ratio of −1 or 1 at an SDR site. In practice, a wider range of values can be observed because gene copy numbers can be highly variable. Alternatively, genes may occur in several different states in terms of copy numbers and a group of diploid individuals in the heterozygous or homozygous state at different copy numbers may result in non-integer log-ratios, as illustrated in Figure S2. Assuming copy number variations are polymorphic within groups, a combination such as 7 copies in *Bamako* samples and 44 copies in *Savanna* samples could produce a log-ratio of −2.65 (−log2(7/44)). Likewise 8 copies in *Bamako* and 3 copies in *Savanna* could produce a log-ratio of 1.41 ( = log2(8/3)). The exact copy numbers for each sample can only be determined after normalizing signals with samples of known copy number, which was not feasible here. Therefore, our data is only informative in identifying sites with copy number variation and cannot be used to determine the exact number of duplicated genes.

**Figure 3:**
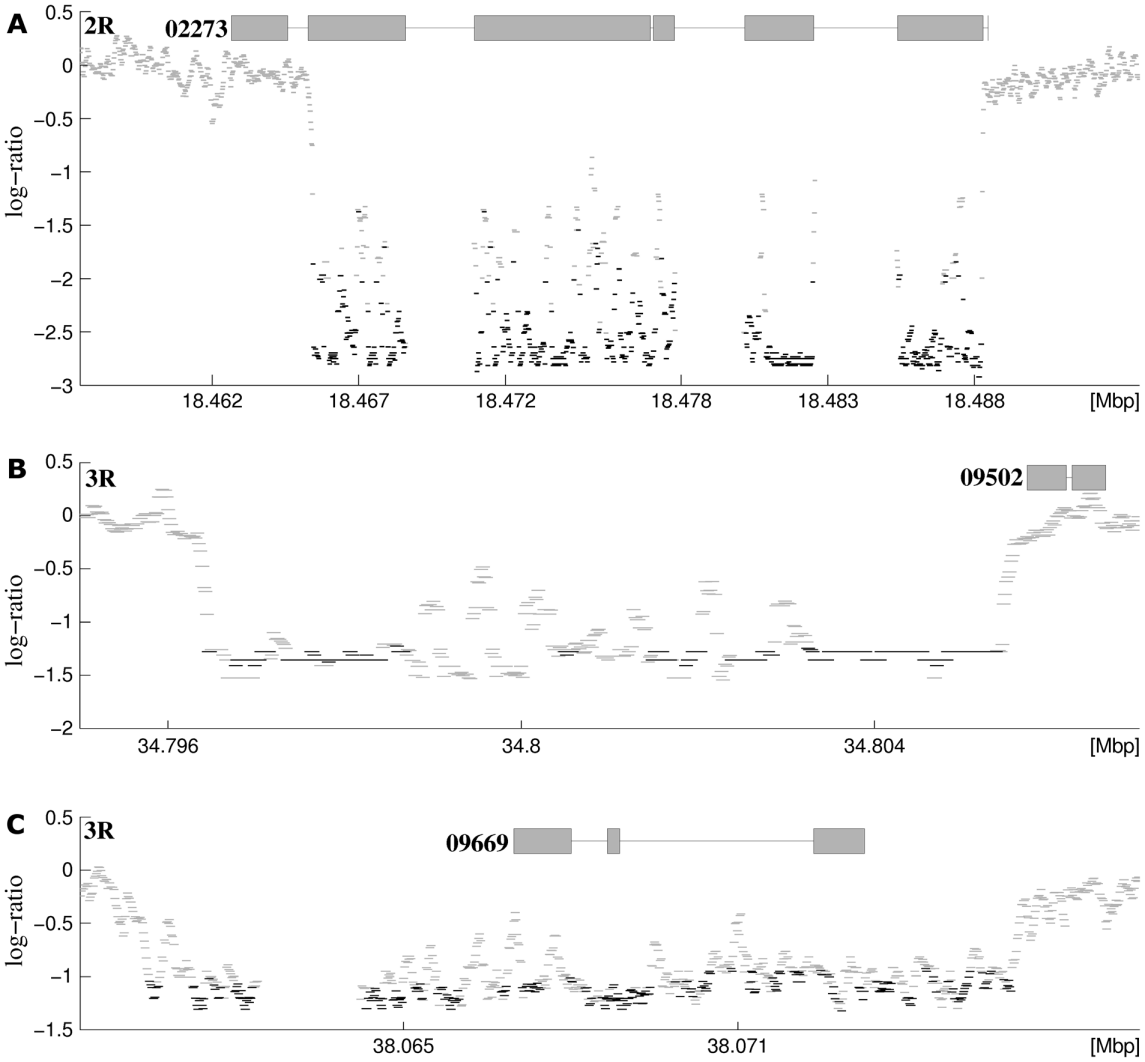
Evidence for gene duplications. **A:** Duplication of gene AGAP002773 on chromosome 2R, located in a hotspot near the 5′ end of inversion 2R*b*; **B:** AGAP009502 located at the 1st hotspot of divergence on the right arm of chromosome 3 (at around 35 Mbp position); and **C:** AGAP009669 located in the 2nd hotspot of divergence on the right arm of chromosome 3 (at around 38 Mbp position). Black bars indicate log-ratios of a SF. Gray bars indicate log ratios of a non-significant probe. Gray boxes and black lines at the top of each graph represent exons and introns respectively. The log-ratio data in the introns of AGAP002273 and upstream of AGAP009669 appear to be missing because genome sequence is unavailable for these regions and consequently no probe was designed for these regions.

Based on this analysis, 205 SDRs showed evidence of 20 gene duplication sites in exon regions, 27 in intron regions and 158 in intergenic regions. Examples of genomic regions where copy number variation was detected are shown in Figure 3. Nine of the 20 gene duplication sites occurred in a single gene of AGAP002273 spread throughout a 23 kbp-long region near the 3' end of the *j* inversion on chromosome 2 (Figure 3A). AGAP002273 is located in the divergence hotspot at the 3' end of the *j* inversion. AGAP009502 (Figure 3B) and AGAP009669 (Figure 3C) are located in the two hotspots on chromosome 3 (Figure 1).

## Discussion

The use of a custom WGTM to compare the genomes of the *Bamako* and *Savanna* chromosomal forms of *A*. *gambiae* detected 0.18% of their genomes to be significantly diverged. The significantly differentiated regions (SDRs) were not randomly distributed throughout the genome, nor restricted to just the autosomes or X chromosome. Rather differentiation was located near the breakpoints of the *j* and *b* inversions, as well as a number of small regions on the X chromosome, particularly around the centromere.

### Chromosome Inversions and “Ecotypic Speciation”

Elevated divergence is expected within inversions for two reasons. First, as per the “ecotypic speciation” model, inversions may contain genes adapted to specific habitats resulting in some degree of spatial isolation between sub-populations. Second, these genes are organized into co-adapted gene complexes [52] that can evolve as a result of restricted recombination that prevents the breakdown of multi-locus genotypes and that may be maintained by association with genes underlying reproductive isolation [6], [53].

Overall, we did not find the density of significantly differentiated features (SFs) to be elevated across 2R inversions of *A. gambiae* as expected. We did find the density of SFs was concentrated near the breakpoints of the *j* and *b* inversions, but this was not the case for the *c* and *u* inversions. The pattern of higher divergence at breakpoints versus near the center of inversions is consistent with results from earlier studies. For example, White et al. [42] compared inverted *jbcu*/*jbcu* with standard arrangements using the Affymetrix *Anopheles*/*Plasmodium* Gene-Chip microarray. They likewise found no evidence of widespread divergence within 2R inversions compared to collinear portions of the genome. An exception in the White et al. [42] study was divergence at the breakpoint of the *u* inversion, a region where we found little divergence using the perfect-match WGTM (see considerations below for discussion of the technical implications of this design). Neafsey et al. [35] likewise found that SNPs differentiating the *Bamako* and *Savanna* forms were more abundant (39/51) at sites located proximal to inversion breakpoints ([35], Supporting Online Materials Table S2, Figure S3).

Earlier work on the genetics of *A. gambiae* populations by Tripet et. al. [54] reported increased microsatellite DNA divergence within the 2R*j* inversion compared with regions outside of this inversion. At first glance, our results appear to contradict this finding. However, on closer examination we found that of the five 2R*j* microsatellite markers they genotyped, three are located near the 3′ breakpoint (one of these overlaps a SDR identified here, Figure S4). The remaining two loci are located near the center of the *j* inversion and one of these lies within a SDR identified in this paper (Figure S4). Thus, the results reported here are consistent with earlier studies which found increased divergence in the *j* inversion is largely restricted to sites near the breakpoints [35], [54]. The genomic location of SDRs in inversion breakpoints correspond to non-coding DNA, with the exceptions of AGAP001487 (2R*j* near the 5' end; Innexin shaking-B encoding gene) and AGAP2273 (2Rb near the 5' end; this is very likely divergence by gene duplication, Figure 3).

### Homogeneity in inversions as a consequence of gene flux

The level of divergence observed between *A. gambiae* chromosomal forms in the current study is an order of magnitude less than the 2.8 Mbp "speciation islands" described between molecular forms (Forest-M *vs* Forest-S forms [8]). This is likely the consequence of there being more contemporary gene flow between *Bamako* and *Savanna* (frequency of hybrids = 0–6.25%, Taylor et al. 2001) than between the Forest-M and Forest-S forms (frequency of hybrids = 0% [20]). This difference in the extent of gene flow between the groups, is further supported by the relatively low F_ST_ between Bamako and Savanna forms (F_ST_ = 0.00559) compared to that between Forest-M and Forest-S forms (F_ST_ = 0.04263 [55]).

Patterns in the distribution of diversity within *A. gambiae* inversions suggest the action of gene flux, whereby crossing-over between alternate inversions occurs *via* gene conversion [56] or double cross-over events [57]. Gene flux results in rates of recombination remaining high in the central region of an inversion, but reduced to almost zero near inversion breakpoints [40], [58], [59]. Studies of gene flux have demonstrated that (1) shorter inversions are expected to have a greater reduction in recombination rate, and (2) reduction of recombination rate is not uniform along an inverted chromosome [60], [61]. Gene flux is only possible when inversions exist in the heterozygous state. The inversions (*j*, *b*, *c* and *u*) do not represent fixed differences between the *Bamako* and *Savanna* forms of *A. gambiae*. It is well established that the *b*, *c* and *u* inversions are shared by both *Bamako* and *Savanna* forms [12]. The *j* inversion, was thought to be fixed, or nearly so [38], but this appears to only be the case in populations along the Niger River (Figure S5). In populations at the village of Founia, just 100 km from the Niger, and populations at sites along the Senegal River in western Mali the *j* inversion is commonly found in the heterozygous state (File S1).

### Genomic divergence on the X chromosome

The most striking result from our study is that it provides the first demonstration of highly diverged X chromosomes within the S molecular form: approximately half of the total SDRs reported here are on the X chromosome. Whilst high X chromosome divergence between the M and S forms has been demonstrated [33], [62], [63], it was unexpected within the S. It is well established that genes involved in reproductive isolation are commonly X-linked [64]–[66], these genes are mostly involved in post-zygotic isolating mechanisms, such as male sterility, which has not been reported between the *Bamako* and *Savanna* forms [13], [14]. Rather, reproductive isolation between the *Bamako* and *Savanna* forms is thought to be maintained by behavioral mechanisms related to mating, as has been described for the M and S molecular forms [13], [14], [38]. Whether genes on the X chromosome are involved in *A. gambiae* pre-mating isolation mechanisms is an avenue worthy of further investigation.

The X chromosome divergence reported here contrast with results reported by Neafsey *et al.* [35], where divergence between *Bamako* and *Savanna* X chromosomes was reported to be minimal. One explanation for this discrepancy centers on the selection of SNPs at the design stage of the SNP-chip employed by Neafsey *et al* [35]. Our WGTM queried all 1092 X-linked genes (both exons and introns) and revealed divergence between *Bamako* and *Savanna* in 101 of these. By contrast, the SNP-chip contains SNPs from selected X-linked genes (139/1092), of which only 77 contain SNPs polymorphic within S form populations. Of the 139 X linked genes queried by the SNP chip, only 11 were found to be divergent using the WGTM (AGAP001050, 1069, 1070, 1073, 1078, 1082, 1084, 1090, 1091,1093 and 13341). The specific SNPs corresponding to these genes represented on the SNP-chip used by Neafsey et al., however, lacked polymorphism within the S form. In other words, 10 of the 11 genes were selected for fixed differences between M and S form rather than for polymorphism within the S form. Consequently, this chip had only 1 of 139 X-linked gene markers that had the potential to be informative for divergence between the *Bamako* and *Savanna* forms. Thus, it is not surprising to find little divergence within S forms using this chip.

Overall, elevated divergence on the X chromosome between both the molecular (M and S) and chromosomal forms (*Bamako* and *Savanna*), suggests that genes on the X chromosome may have a common role in speciation within *A. gambiae*. This is further supported by the finding that among the X-linked genes distinguishing the *Bamako* and *Savanna* forms are a heat shock protein (AGAP001070) and cytochrome P450 (AGAP001076), both of which were earlier identified to contain fixed differences between the M and S molecular forms [8]. This supports the possibility that a common X-linked SDR modulates reproductive isolation both between the M and S molecular forms and the *Bamako* and *Savanna* chromosomal forms and that these may be widely shared among *A. gambiae* sub-taxa.

### Copy number variation as a mechanism for divergence between *Bamako* and *Savanna*

Assessment of copy number variation is not possible through conventional sequencing or microsatellite studies, and thus has not received much attention in previous population genetic studies examining divergence within *A. gambiae* (but see [15]). Gene duplication can promote speciation by creating functional divergence between two genes created by a duplication event [48]. Several reports have demonstrated duplicated genes evolving completely new functions in various vertebrate species and in *Drosophila* [44]–[49], [67].

The high number of gene duplication sites in the intergenic regions (158, File S2) was unexpected. Our data suggests that gene duplication may have an important role in genomic divergence between the *Bamako* and *Savanna* forms, as illustrated in Figure 3. How copy number variation influences divergence (e.g. by altering gene expression) in *A. gambiae* and whether it is important for divergence and reproductive isolation between other *A. gambiae* subgroups, remains to be seen. Nonetheless, our findings indicate that assessment of copy number variation in future studies of divergence in this taxon would be worthwhile.

### Limitations

It is important to consider the results of this study in light of two technical limitations, which may explain why we did not detect fixed differences between the *Bamako* and *Savanna* forms. First, palindromic sequences longer than 25 bp would not be easily detected using the Ag-WGTM because any fragment with complimentary sequence will hybridize to a 25 bp-long probe, regardless of direction. The other technical consideration concerns the microarray design. The WGTM used here contains perfect-match probes only. The effect of this feature, in terms of signal detection, is illustrated in Figure S6. Our WGTM may fail to detect divergence between the *Bamako* and *Savanna* forms in cases where the probes are not only different between the two forms, but also different from the reference PEST genome sequence.

Another important consideration is the high variability in space and time of *A. gambiae* populations. Figure S1A shows probe hybridization intensities for the two groups of samples at the 5′ end of the *j* inversion, a region where significant differentiation was detected. Visual inspection of the samples collected at the same time and from sites only 25 km apart appear different (though not statistically). Further evidence of this effect is suggested by the distribution of the *j* inversion among *A. gambiae* populations along the Niger *versus* the Senegal Rivers with respect to known chromosomal forms elsewhere in Africa.

The origin of specimens used for comparative genomics may have been a factor in explaining why our results differ from earlier reports. Neafsey et al. [35] used specimens from different geographic locations in Mali (Kela, Banambani, and Bancouma) and these specimens were collected at different times than the samples used here. Geographic or temporal variation either through selection or drift thus may have played a role in our detecting genomic differentiation on the X chromosome. In addition, Neafsy et al. used heterogenous inversion genotypes, both for the *Bamako* and *Savanna* forms, whereas we selected two specific inversion arrangements (*jbcu* and *+*) for our comparison.

### Conclusion

Identifying the genes underlying reproductive isolation has long been of interest to evolutionary biologists. Growing evidence suggests that divergence between genomes with gene flow can be attributed to a relatively small number of genes, often located on the X chromosome and at sites where recombination is restricted (e.g. breakpoints of inversions). Consistent with expectations that inversions are involved in the divergence process, we found elevated genomic divergence between *Bamako* and *Savanna* in the regions carrying inversions. However, divergence was not observed throughout the inversions, rather it is restricted to sites near the breakpoints. This suggests that through gene flux, recombination (gene flow) can occur via double crossing over events between standard and inverted chromosomal arrangements.

Strikingly, the overall highest divergence was found on the X chromosome. Divergence on the X has been reported between the molecular forms in *A. gambiae*, but, before now, not within them. Interestingly, some X-linked genes previously reported to contain fixed differences between the Forest-M and Forest-S forms [8] were also found to be significantly diverged between the *Bamako* and *Savanna* forms. This suggests that there exist some common X-linked genes modulating reproductive isolation and that these are widely shared among *A. gambiae* sub-taxa.

Understanding the extent of reproductive isolation among populations of this species and the mechanisms underlying it is significant because this system has become widely studied as a model for sympatric speciation and because of the significance to understanding the genetic structure of populations to applied research aimed at malaria control (e.g. association mapping studies of *Plasmodium* susceptibility, insecticide resistance and to the development and application of genetically modified mosquitoes for malaria control). Among the genes that overlap with SDRs, 94% encode proteins of unknown function. This makes it difficult to form a detailed hypothesis of what is driving genomic differentiation between the two forms. Future studies on functional genomics of this species are critical to understanding the evolution of reproductive isolation and adaptation among populations of this species.

## Materials and Methods

### Sample collection and preparation

Half-gravid female *A. gambiae* mosquitoes were collected before noon from indoor human sleeping areas by mouth aspiration from 15 villages in Mali (Figure S5). Ovaries from these females were preserved in modified Carnoy's solution (3 parts glacial acetic acid: 1 part ethanol) and the remainder of each mosquito was preserved in 70% ethanol in the field. Chromosomes were scored at the five major chromosomal inversions: 2L *a*, 2R *j*, *b*, *c* and *u*, using standard cytogenetic protocols outlined by Hunt [68]. The karyotype of each individual was designated using standard nomenclature, where the standard (non-inverted) arrangement is denoted "+/+" and inverted arrangement denoted by the name of the inversion e.g. “*j*/*j*”. The *Bamako* and *Savanna* forms each consist of a number of unique karyotypes. We specifically selected homokaryotype 2R+ from the *Savanna* form and homokaryotype 2R*jbcu* from the *Bamako* form to maximize the potential differentiation between the forms due to inversions (Table 1). All specimens were homozygous for the 2L*a* inversion (*a/a*) and all were of the S molecular form as determined using the protocol of Favia et al. [69].

Genomic DNA from the remainder of each individual mosquito (head, thorax and remainder of abdomen) was extracted using a Qiagen Biosprint 96 machine and the standard Qiagen Blood and Tissue kit and protocol. Molecular form for each specimen was determined using an RFLP method developed by Fanello *et al.* [18] and/or a PCR method developed by Favia *et al.* [69].

Samples for comparative genomics were selected from sites where the *Bamako* and *Savanna* forms are found in sympatry (Figure S5). The *Savanna* form with homokaryotype 2R+ were rare, which meant that despite having access to 1,680 S form samples collected in Mali from 2002–2006, only seven specimens met our criteria. Thus, the only option available to us was to include from multiple locations in different time points. The sample size for microarray hybridization experiments is determined by performing a priori power analysis given 13 million probes to achieve power of 0.95 after multiple comparison adjustment of non-parametric tests such as Wilcoxon rank sun test [70].

Seven *Bamako* and seven *Savanna* samples were hybridized to custom *A. gambiae* WGTMs (Affymetrix, Santa Clara, CA). Sample IDs for the selected mosquitoes and related molecular data are presented in Table 1. The IDs are a concatenation of collection site, collection date and tube index number, and are the identification keys for individual level data publicly accessible from the *PopI* database (https://grassi2.ucdavis.edu). A map of the collection sites was compiled by projecting GPS locations onto the GlobCover Land Cover Types (Figure S5) as described in [55].

### DNA labeling and hybridization to microarray

Due to the limited amount of sample DNA, we chose the random priming method over a restriction enzyme method for DNA labeling. We used the Invitrogen BioPrime DNA labeling system to label genomic DNA with 300 ng of template. For each reaction, we added 60 µl of 2.5X random primer solution to the DNA, and added distilled water to bring the total volume to 132 µl. The mixture was then denatured at 95°C for 5 minutes and cooled on ice. 15 µl of a 10X dNTP mixture and 3 µl of Klenow Polymerase from the labeling kit were added to this mixture which was incubated at room temperature overnight. The following day, labeled products were purified using 15 µl of 3 M sodium acetate and 400 µl of 100% ethanol that had been cooled to 4°C. Following mixing, each reaction was held at −80°C for 15 minutes and subsequently centrifuged at 15,000 g for 10 minutes. After decanting the liquid, 500 µl of cooled 75% ethanol was added and mixed thoroughly. This solution was centrifuged at 15,000 g for an additional 10 minutes. After decanting the alcohol, the DNA was air dried and resuspended in 100 µl of distilled water. Quality of labeling was assessed by running 3–5 µl of labeled product on a 3% agarose gel. Each sample was hybridized to the two chips containing 13 million probes using the standard GeneChip® hybridization protocol provided by Affymetrix® by the UC Davis Expression Analysis Core facility.

### Microarray data analysis

Affymetrix® Tiling Analysis Software (TAS) version 1.1 and Tiling Analysis Command Line Software (Tilecore) release 2010-04-21 were used to perform a two sample probe analysis. The analysis performs a Wilcoxon rank-sum test comparing the log-transformed signal intensities between the two groups (*Bamako* versus *Savanna*) to compute p-values and log-ratios. All samples were quantile normalized together. The Hodges-Lehmann estimator of fold enrichment [71] was also computed. This is the median pairwise log-ratio of the intensity values used for each Wilcoxon rank-sum test. A bandwidth of 77 was used for analysis, except for the high probe density region near the centromere on the X chromosome, where we used a bandwidth of 22. This is equivalent to a window size ( = two times the bandwidth +1) of 155 bp and 45 bp respectively, which results in a robust median value of 9 probes per window. By using this adjusted window size for the high resolution region on the X chromosome, we minimize the overestimation of significance value. For further information, refer to the TAS manual available at http://www.affymetrix.com/support/developer/downloads/TilingArrayTools/TileArray.pdf.

The false discovery rate was calculated using *Q-value* software [51] available at http://genomics.princeton.edu/storeylab/qvalue/index.html. Scripts were developed in Python version 2.6.2 for data extraction, collation, and file conversion. Integrated Genome Browser version 6.3 and Matlab version 6.0.0.88 were used for visualization. The hit density was defined as the fraction of significant probes divided by the number of available probes within a region (window). We used window sizes of 200 kbp and 1 Mbp with 50% overlap between windows for plotting hit density.

Determining the size threshold for detecting gene/exon duplication is not straightforward because the length of an exon can vary from less than 100 bp to over 14,000 bp in *A. gambiae* according to the AgamP3 annotation. Changes in regulatory regions such as enhancers and insulators could also produce phenotypic differences. In Metazoan genes, regulatory regions vary from 300 bp to 2 kb in length [72]. Here, we used 300 bp as a threshold to examine whether any of the SDRs identified from our earlier analyses (File S2) were potential gene/exon duplication sites.

### Annotation Data

We present our results based upon the AgamP3 58.3 k annotation version from Ensembl. The *ReAnoCDS* annotation [73] was not used because it is based on an older version of the genome sequence which is different from the one used to design the *Ag-*WGTM. For instance, the *ReAnoCDS* X chromosome sequence is 2.3 Mbp shorter than the current version.

### Data accessibility

A detailed list of SFs with their genome coordinates, p-value, log-ratio, nearest Ensembl gene ID, and the distance from that gene to the probes is provided in File S2. A query interface for this information is available on *PopI*: https://grassi2.ucdavis.edu/under "AgArray" Open Projects. Individual CEL files and probe library files are also available on *PopI*.

### Ethics statement

No specific permits were required for the described field studies.

## Supporting Information

Figure S1 **Illustration of individual intensity plots for selected regions.**
(PDF)

Figure S2 **Illustration of a hypothetical scenario of copy number variation.**
(PDF)

Figure S3 **Location of genes reported to be diverged between** ***Bamako*** **and** ***Savanna*** **forms in relation to inversions on 2R.**
(PDF)

Figure S4 **Illustration of 2R** ***j*** **microsatellite marker locations that showed significant divergence within 2R** ***j*** **inversion in relation to divergence observed in this study.**
(PDF)

Figure S5 **A map of 2R** ***j*** **inversion distribution in Mali.**
(PDF)

Figure S6 **Illustration of probe hybridization characteristics in perfect match microarray.**
(PDF)

File S1 **Individual karyotypes of collected specimens from 15 sites shown in** **Figure 1** **.**
(XLSX)

File S2 **SDR list in tabular format.**
(XLSX)
