# Supplementary material for: Chromosome Inversions, Genomic Differentiation and Speciation in the African Malaria Mosquito Anopheles gambiae
Source: PLoS One. 2013 Mar 20;8(3):e57887. doi: 10.1371/journal.pone.0057887 (PMC3603965; doi:10.1371/journal.pone.0057887)
Supplement: Figure S1 — Illustration of individual intensity plots for selected regions. (PDF) [file pone.0057887.s001.pdf]

Figure S1. Magnified view of individual intensity readings for probes along a 10kbp segment of chromosome 2R and 4kbp segment of the X chromosome. Each row represents an individual sample. The designations, A1, A2, etc. correspond to data presented in Table 1 of the main text.

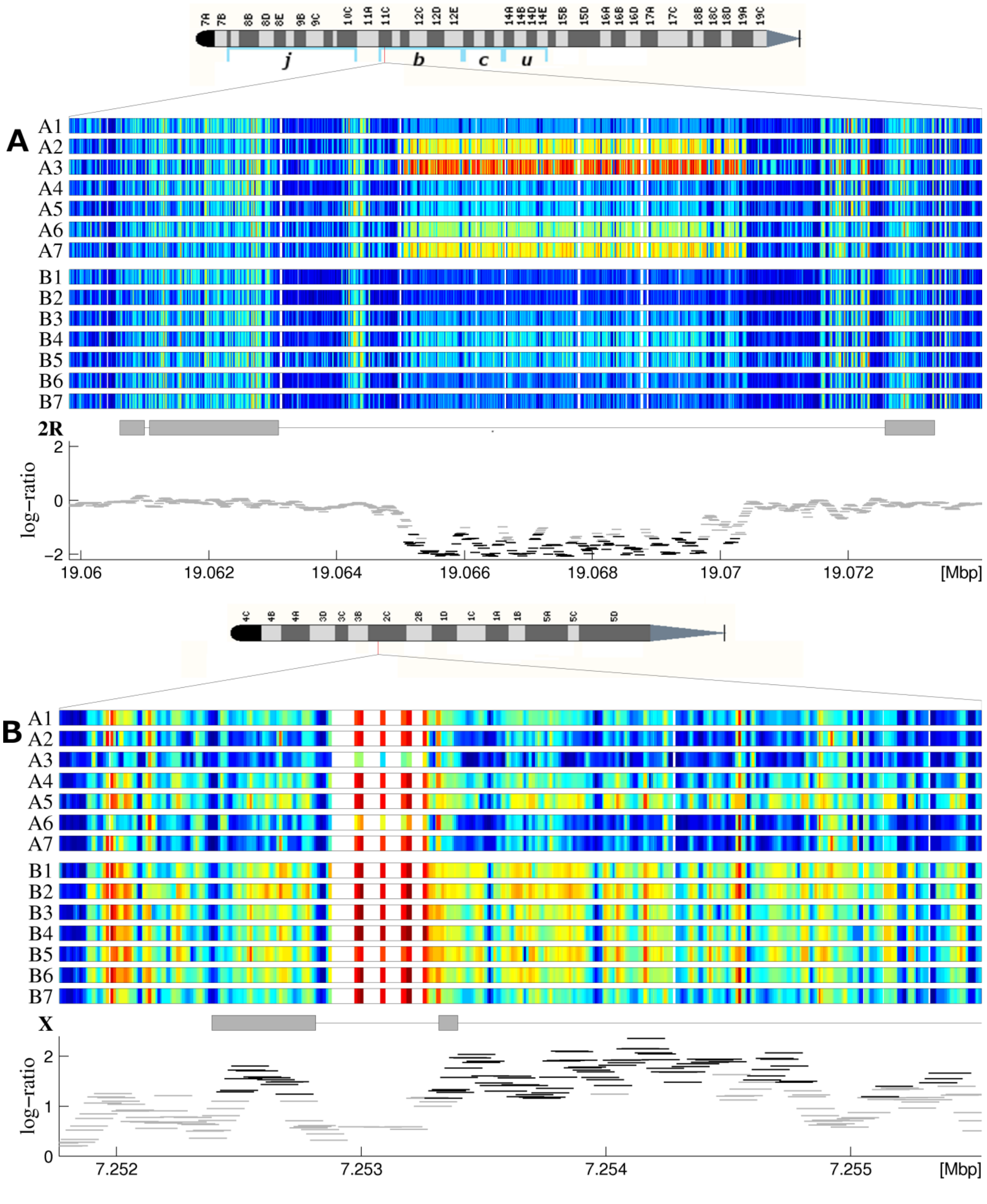

Colors closer to dark blue indicate weaker hybridization to a probe and colors closer to red indicates stronger hybridization. The X axis on the log-ratio plots indicate genome coordinate on the chromosome. White color indicates a gap where probes were not placed on the chip due to design constraints. Log-ratio values for significantly differentiated regions are shown as black lines, whereas undifferentiated regions are shown as grey lines. **A**: area proximal to the 5' breakpoint of the 2Rb inversion. This segment of more than 4kb overlaps with a gene, AGAP002309. Exons of this gene are marked in gray boxes and the line connecting exons indicates intron regions. It can be seen that *Bamako* forms (B1-7) have poorer hybridization than *Savanna* form (A1-7) in the intron region. **B**: a 4kbp section of a transcript, AGAP000399-RB on the X chromosome.

Assessment of individual intensity values shown above indicates that a 4kbp-long deletion may be present in the intron sequence of the AGAP002309 gene located in the 2Rb inversion in the *Bamako* form samples (B1-7) and in some *Savanna* form samples (A1, 4-5). A 1.5kb region covering exon 6 and the adjacent intron of the AGAP000399-RB transcript on the X chromosome shows stronger hybridization in the *Bamako* forms relative to *Savanna* forms (Figure S3B). This is likely due to an exon duplication rather than sequence polymorphism.

Figure S1A shows greater hybridization intensity for Savanna form samples, while Figure S1B shows the opposite. It is important to note that these figures display a very narrow region (<10kb) of chromosomes 2 and 3 and are not typical of genome wide hybridization intensities patterns overall.
