# Supplementary material for: Chromosome Inversions, Genomic Differentiation and Speciation in the African Malaria Mosquito Anopheles gambiae
Source: PLoS One. 2013 Mar 20;8(3):e57887. doi: 10.1371/journal.pone.0057887 (PMC3603965; doi:10.1371/journal.pone.0057887)

Figure S2. A hypothetical scenario where copy number variation can result in non-integer log-ratios. A single gene duplication event results in a log-ratio of 1 or -1. However a non-integer log-ratio is possible if copy number variation (CNV) occurs in a few different states or CNV genotypes. A log ratio of -2.65, for instance, requires a copy number ratio of 1:6.3. This can be achieved, for example, by having *Bamako* form fixed with one copy and *Savanna* forms with variable genotype of 6/6, 6/7, or 7/7, as illustrated below.

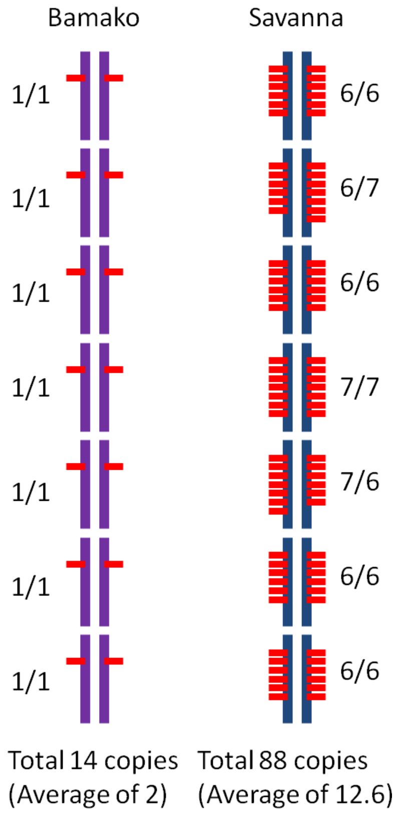

Supplement: Figure S2 — Illustration of a hypothetical scenario of copy number variation. (PDF) [file pone.0057887.s002.pdf]
