# Supplementary material for: Chromosome Inversions, Genomic Differentiation and Speciation in the African Malaria Mosquito Anopheles gambiae
Source: PLoS One. 2013 Mar 20;8(3):e57887. doi: 10.1371/journal.pone.0057887 (PMC3603965; doi:10.1371/journal.pone.0057887)
Supplement: Figure S3 — Location of genes reported to be diverged between Bamako and Savanna forms in relation to inversions on 2R. (PDF) [file pone.0057887.s003.pdf]

Figure S3. A: Genes reported to be diverged between *Bamako* and *Mali-S* (*Savanna*) form by Neafsey et al. [1] are marked in red. Inversion j, b, c, and u are marked with blue - blocks indicates uncertainty in starting and end of breakpoints. Most of the divergence was observed near breakpoints of inversions except for the 3' end of inversion *c* and 5' end of inversion *u*. **B:** Genes reported to be diverged between *Bamako* and *Savanna* form in the right arm of chromosome 2 by Neafsey et al. [1]

A

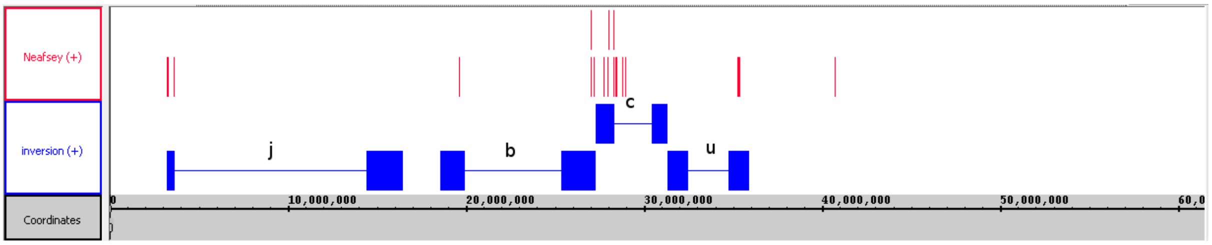

B

Chromosome	gene id	gene start	gene end	location relative to inversion
2R	AGAP001333	3253875	3257684	5' end of 2R j
2R	AGAP001334	3260240	3261465	5' end of 2R j
2R	AGAP001335	3262688	3262290	5' end of 2R j
2R	AGAP001337	3308742	3311392	5' end of 2R j
2R	AGAP001338	3313345	3317264	5' end of 2R j
2R	AGAP001367	3590764	3632244	5' end of 2R j
2R	AGAP001368	3634518	3645862	5' end of 2R j
2R	AGAP002325	19610696	19658751	5' end of 2R b
2R	AGAP002779	27051549	27053652	3' end of 2R b
2R	AGAP002780	27053652	27054296	3' end of 2R b
2R	AGAP002781	27054619	27059551	3' end of 2R b
2R	AGAP002782	27059818	27078390	3' end of 2R b
2R	AGAP013378	27062359	27063674	3' end of 2R b
2R	AGAP013010	27064765	27067922	3' end of 2R b
2R	AGAP002783	27181699	27189160	3' end of 2R b
2R	AGAP002784	27191455	27206429	3' end of 2R b
2R	AGAP002802	27742225	27762549	5' end of 2R c
2R	AGAP002824	27997113	28036208	5' end of 2R c
2R	AGAP002825	28007274	28014861	5' end of 2R c
2R	AGAP002846	28294800	28295651	5' end of 2R c
2R	AGAP013388	28298607	28295651	5' end of 2R c
2R	AGAP002847	28299667	28300338	5' end of 2R c
2R	AGAP002848	28300478	28301113	5' end of 2R c
2R	AGAP002849	28302217	28302760	5' end of 2R c
2R	AGAP002850	28304726	28305246	5' end of 2R c
2R	AGAP002851	28305680	28306258	5' end of 2R c
2R	AGAP002852	28307397	28309299	5' end of 2R c
2R	AGAP002853	28309739	28310362	5' end of 2R c
2R	AGAP002854	28311004	28311650	5' end of 2R c
2R	AGAP002855	28313122	28314070	5' end of 2R c
2R	AGAP002856	28314102	28314668	5' end of 2R c
2R	AGAP002858	28322913	28389385	5' end of 2R c
2R	AGAP002859	28420677	28511124	5' end of 2R c
2R	AGAP002888	28822444	28860915	5' end of 2R c
2R	AGAP002896	28997201	29014636	5' end of 2R c
2R	AGAP003301	35250358	35339805	3' end of 2R u
2R	AGAP003302	35342744	35344387	3' end of 2R u
2R	AGAP003303	35351562	35357083	3' end of 2R u
2R	AGAP003304	35362528	35363334	3' end of 2R u
2R	AGAP003631	40725964	40738059	outside of inversion

REFERENCES

1. Neafsey DE, Lawniczak MK, Park DJ, Redmond SN, Coulibaly MB, et al. (2010) SNP genotyping defines complex gene-flow boundaries among African malaria vector mosquitoes. Science 330: 514-517.
