# Supplementary material for: Chromosome Inversions, Genomic Differentiation and Speciation in the African Malaria Mosquito Anopheles gambiae
Source: PLoS One. 2013 Mar 20;8(3):e57887. doi: 10.1371/journal.pone.0057887 (PMC3603965; doi:10.1371/journal.pone.0057887)
Supplement: Figure S4 — Illustration of 2R j microsatellite marker locations that showed significant divergence within 2R j inversion in relation to divergence observed in this study. (PDF) [file pone.0057887.s004.pdf]

Figure S4. Microsatellites genotyped by Tripet et al. [1] that showed significant divergence within the *j* inversion. Microsatellite markers in relation to the genomic coordinates (red line) and significant features (SFs) indicated in this study (blue line) are illustrated. Chromosome 3 regions are not shown because none of the five microsatellites overlapped with SFs.

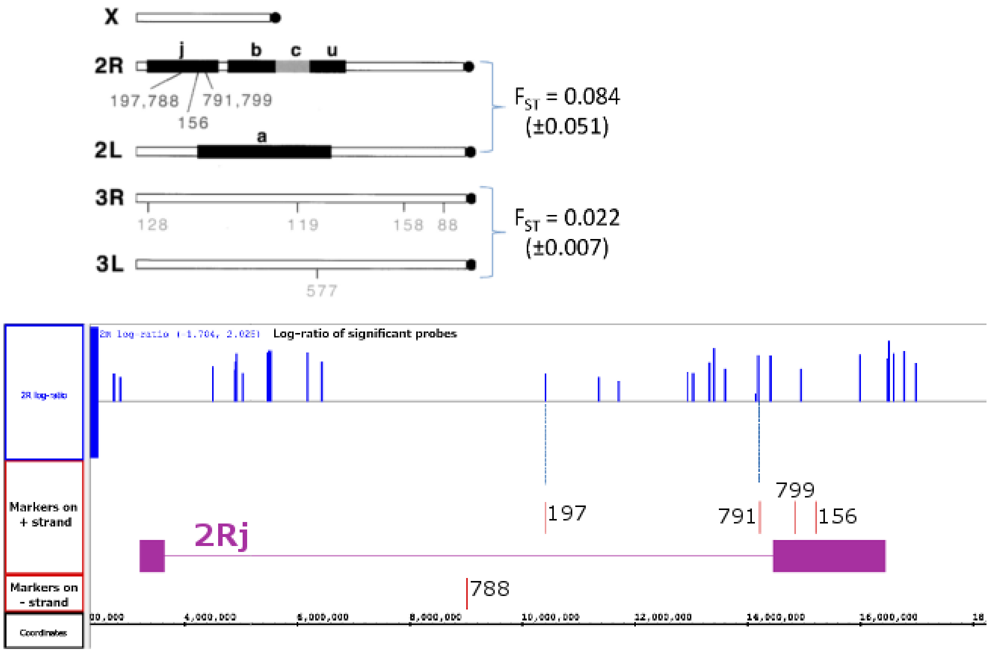

REFERENCES

1. Tripet F, Dolo G, Lanzaro GC (2005) Multilevel analyses of genetic differentiation in *Anopheles gambiae* s.s. reveal patterns of gene flow important for malaria-fighting mosquito projects. Genetics 169: 313-324.
