# Supplementary material for: Chromosome Inversions, Genomic Differentiation and Speciation in the African Malaria Mosquito Anopheles gambiae
Source: PLoS One. 2013 Mar 20;8(3):e57887. doi: 10.1371/journal.pone.0057887 (PMC3603965; doi:10.1371/journal.pone.0057887)
Supplement: Figure S5 — A map of 2R j inversion distribution in Mali. (PDF) [file pone.0057887.s005.pdf]

FIGURE S5. 2Rj inversion distribution in Mali

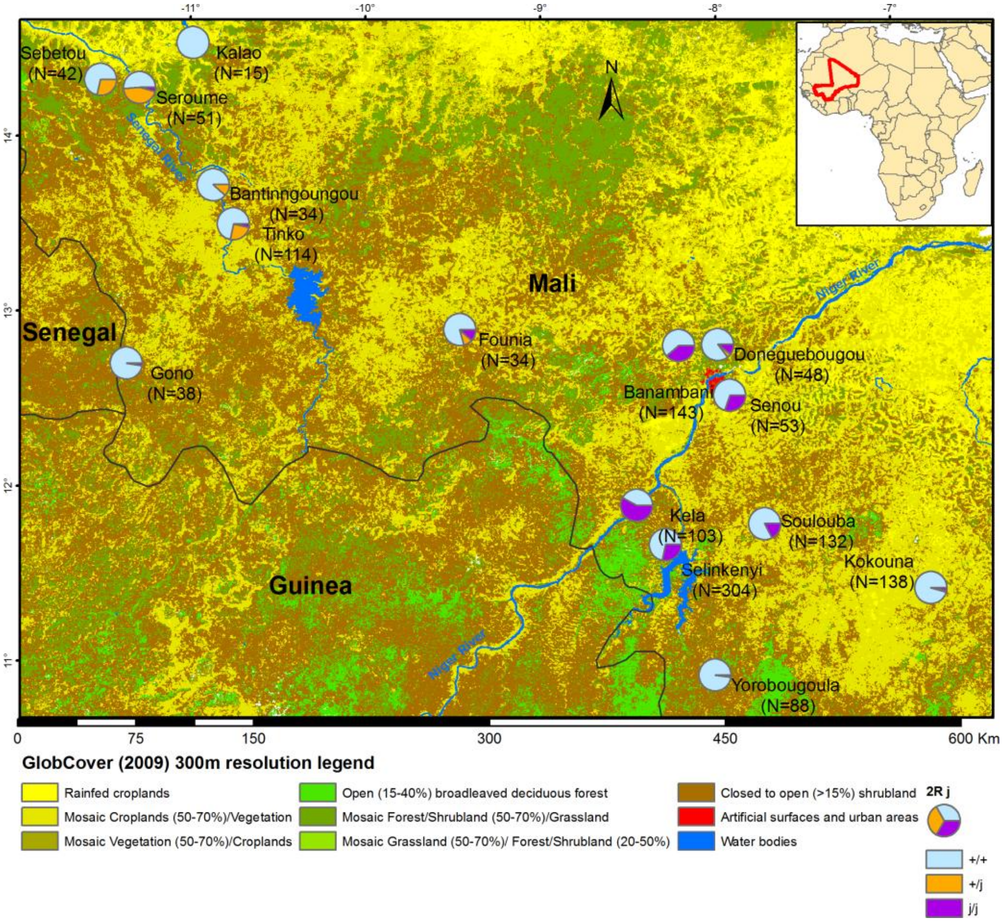

The 2R *j* inversion polymorphism in Mali shows two mating patterns in different parts of the species range in this country. At sites along the Senegal River (e.g. villages of Sebetou, Seroume, Bantinngoungou, and Tinko), 2R *j* inversion heterozygotes are commonly found and 2R *j* karyotypes are in Hardy-Weinberg expectation (HWE). On the other hand, at sites along the Niger River and its tributaries (e.g. villages of Banambani, Doneguebougou, Senou, Kela, Selinkenyi, Soulouba, Yorobougoula, Kokouna), a severe deficiency of 2R*j* heterokaryotypes are observed and 2R*j* karyotypes are not in HWE.

In the literature the *Bamako* form includes three genotypes, *jcu*/*jcu*, *jcu*/*jbcu*, and *jbcu*/*jbcu*, all homozygous for *j* [1]. Other individuals carrying the 2R *j* inversion but not *c* and *u* inversions such as *jbd*/*jbd*, and *jb*/*b*, commonly found along the Senegal River, cannot be classified under the current definitions for chromosomal forms. 94% of the 2R *j* homozygotes along the Niger River are *Bamako* forms, while no *Bamako* forms are found along the Senegal River. Karyotypes of each individual specimen represented in this figure are provided in File S1.

REFERENCES

1. Touré YT, Petrarca V, Traoré SF, Coulibaly A, Maiga HM, et al. (1998) The distribution and inversion polymorphism of chromosomally recognized taxa of the *Anopheles gambiae* complex in Mali, West Africa. Parassitologia 40: 477-511.
