# Supplementary material for: Chromosome Inversions, Genomic Differentiation and Speciation in the African Malaria Mosquito Anopheles gambiae
Source: PLoS One. 2013 Mar 20;8(3):e57887. doi: 10.1371/journal.pone.0057887 (PMC3603965; doi:10.1371/journal.pone.0057887)
Supplement: Figure S6 — Illustration of probe hybridization characteristics in perfect match microarray. (PDF) [file pone.0057887.s006.pdf]

Figure S6. Probe hybridization characteristics in the *A. gambiae* Whole Genome Tiling Microarray

Probes on the *A. gambiae* whole genome tiling microarray used in this study are perfect-match only probes. Thus, any mutation from the reference sequence (*A. gambiae* PEST strain) will be washed away during the hybridization step, resulting in a lack of signal in the corresponding probe [1]. Even if the *Bamako* and *Savanna* forms have mutations that are different from each other, such divergence will not be detected on a perfect-match only microarray if both mutations differ from the reference sequence, the PEST strain in this case.

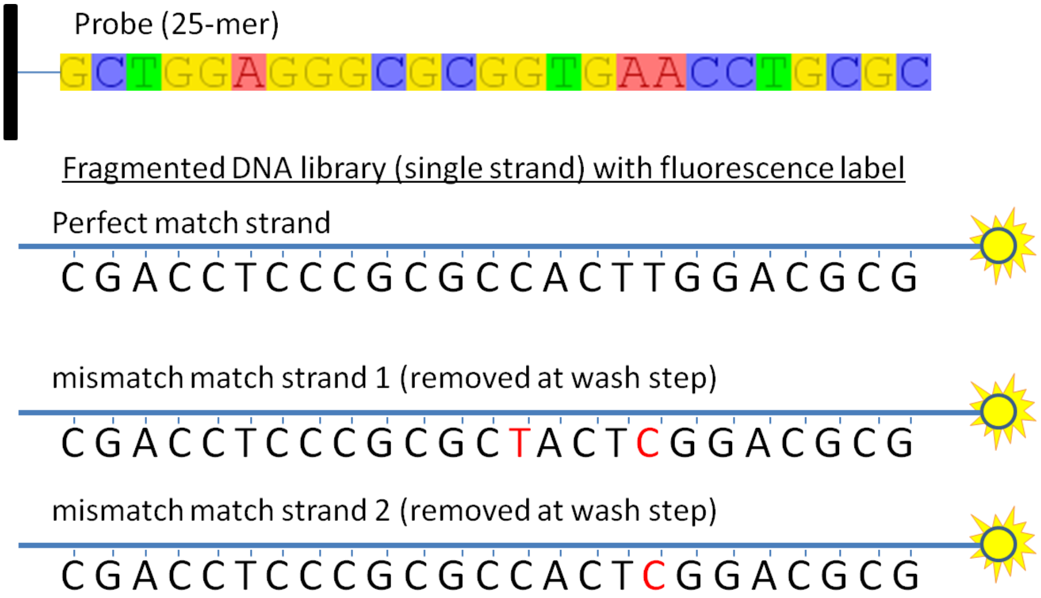

REFERENCES

1. Lipshutz RJ, Fodor SP, Gingeras TR, Lockhart DJ (1999) High density synthetic oligonucleotide arrays. Nat Genet 21: 20-24.
